# Synergistic interplay of Gβγ and phosphatidylinositol 4,5-bisphosphate dictates Kv7.4 channel activity

**DOI:** 10.1007/s00424-016-1916-4

**Published:** 2016-12-15

**Authors:** Oleksandr V. Povstyan, Vincenzo Barrese, Jennifer B Stott, Iain A Greenwood

**Affiliations:** Vascular Biology Research Centre, Molecular and Clinical Sciences Institute, St George’s, University of London, London, SW17 0RE UK

**Keywords:** Potassium channel, KCNQ, PIP_2_, G-protein βγ, Ion channel regulation

## Abstract

Kv7.4 channels are key determinants of arterial contractility and cochlear mechanosensation that, like all Kv7 channels, have an obligatory requirement for phosphatidylinositol 4,5-bisphosphate (PIP_2_). βγ G proteins (Gβγ) have been identified as novel positive regulators of Kv7.4. The present study ascertained whether Gβγ increased Kv7.4 open probability through an increased sensitivity to PIP_2_. In HEK cells stably expressing Kv7.4, PIP_2_ or Gβγ increased open probability in a concentration dependent manner. Depleting PIP_2_ prevented any Gβγ-mediated stimulation whilst an array of Gβγ inhibitors prohibited any PIP_2_-induced current enhancement. A combination of PIP_2_ and Gβγ at sub-efficacious concentrations increased channel open probability considerably. The stimulatory effects of three Kv7.2-7.5 channel activators were also lost by PIP_2_ depletion or Gβγ inhibitors. This study alters substantially our understanding of the fundamental processes that dictate Kv7.4 activity, revealing a more complex and subtle paradigm where the reliance on local phosphoinositide is dictated by interaction with Gβγ.

## Introduction

The Kv7 family of potassium channels (Kv7.1–Kv7.5) are crucial determinants of cardiac, neuronal, cochlear and vascular function [25, 26]. As such, defining the mechanisms that control how Kv7 channel activity is regulated is crucial. It is acknowledged that Kv7 channels have an obligatory requirement for phosphatidylinositol 4,5-bisphosphate (PIP_2_) [4, 12, 18], but we recently described how the Kv7.4 channel, important for normal vascular function [6, 7, 15], requires G-protein βγ subunits (Gβγ) for its voltage-dependent activity [27]. Few ion channels are directly regulated by Gβγ with the best studied being the G protein coupled inwardly rectifying potassium (GIRK) channel that controls electrical excitability in neurons and cardiac cells [9–11, 19]. This channel is comprised of Kir3.1–3.4 subunits and Gβγ enhance open probability by stabilizing the interaction of PIP_2_ [9, 13, 20]. We speculated whether the stimulatory effects of Gβγ on Kv7.4 were due to an interaction with PIP_2_ analogous to GIRK channels. As such, Gβγ would be ineffective under conditions where PIP_2_ levels were reduced. However, the study revealed a level of regulation far more complex and provides the first account of a synergistic regulation of Kv7.4 channels by Gβγ and PIP_2_.

## Methods

### Cell culture

Human embryonic kidney cells (HEK293) stably transfected with Kv7.4 were maintained in modified Eagles’ medium containing 10% foetal calf serum, 1% penicillin/streptomycin, 1% non-essential amino acids, 1% l-glutamine and 1% sodium pyruvate. For experiments, cells were detached by brief trypsin treatment. HEK Kv7.4 cells were plated on 13-mm coverslips in an external physiological salt solution (PSS) containing (mM): KCl 6, NaCl 120, MgCl_2_ 1.2, CaCl_2_ 2.5, d-glucose 12 and HEPES 10, pH was adjusted to 7.35 with NaOH. Cells were left on cover slips for 30 min at room temperature and stored at 4 °C for up to 8 h.

### Whole-cell electrophysiology

Macroscopic transmembrane ionic currents of HEK293 Kv7.4 cells were recorded using standard amphotericin B (300 μg/ml) perforated-patch techniques in voltage-clamp mode. In some experiments, the ruptured whole-cell patch-clamp technique was used for intracellular perfusion of active Gβγ subunits. Patch pipettes were fire-polished and had a resistance of 4–8 MΩ when filled with the pipette solution of the following composition (mM): KCl 126, MgCl_2_ 1.2, HEPES 10 and EGTA 0.5, and pH was adjusted to 7.2 with KOH. Na_2_ATP (1 mM) was added to the pipette solution for the ruptured whole-cell experiments. Cells were held at −60 mV and current amplitude was monitored by application of test pulse to +20 mV. To generate current-voltage relationships, a voltage step protocol was used from a holding potential of −60 mV, testing a range of voltages from −90 to +40 mV in 10-mV increments at 15-s intervals. Drugs were applied in the external solution using a bath perfusion system, except for Gβγ subunits which were included in the pipette solution.

### Single channel electrophysiology

Single-channel activity of Kv7.4 currents expressed in HEK293 cells was recorded in voltage-clamp mode using inside-out patch configuration in an external solution of the following composition (mM): KCl 165, HEPES 5 and EGTA 10, and pH was adjusted to 7.2 with NaOH. Patch pipettes were fire-polished and had a resistance of around 20 MΩ when filled with PSS as a pipette solution. Cells were voltage clamped at 0 mV. Cell-attached patch configuration was used in some experiments. In this case, PSS was used for both, pipette and bath solutions, and the cells were recorded at −50 mV, so the expected membrane potential under the patch is around −6 mV (assuming resting membrane potential of −56 mV [21]). All single-channel current records were filtered at 0.1 kHz using a Frequency Devices 9002 digital filter with 8-pole low-pass Bessel filter and acquired at 1 kHz (Axopatch 200B 4-pole low-pass Bessel filter). Current amplitudes were calculated from idealized traces of ≥180 s in duration using 50% threshold method using pClamp 9.0 software. Events lasting less than 6.664 ms (2 × rise time for a 100 Hz, −3 db, low-pass filter, [8]) were excluded from the analysis to maximize the number of channel openings reaching their full current amplitude. Channel activity was expressed as NPo, which was calculated automatically and reported by the pClamp 9.0 software under “event statistics” after completion of the single-channel search procedure applied to the idealized traces ≥180 s in duration at each condition. All whole-cell and single-channel current recordings were made using AXOpatch 200B amplifier (Axon Instruments) at room temperature. Electrical signals were generated and digitized using a Digidata 1322A hosted by a PC running pClamp 9.0 software (Molecular Devices). Drugs were applied in the external solution using a push-pull system. All electrophysiological data were analysed and plotted using pClamp 9.0, MicroCal Origin 6.0 (MicroCal software, Northampton, MA, USA) and GraphPad Prism (GraphPad Software, Inc., La Jolla, CA, USA). PIP_2_ and βγ G proteins were applied at a range of concentrations to inside-out excised patches to determine the sensitivity of the Kv7.4 channel to these modulators. However, it was not usually possible to hold the patch long enough to do a full concentration-response and saturating responses were not achieved. Data are therefore accumulated from a number of patches and fitted by a sigmoidal curve to give an approximate estimate of sensitivity. Values for half maximal stimulation taken from these fits are quoted in the text with the caveat that saturation of channel enhancement was not seen with either molecule.

### In-cell western blot

In-cell western blot experiments were performed as described elsewhere [5]. HEK293 cells stably expressing Kv7.4 were grown in 96-well plates for 24 h and incubated with different drugs as indicated. After treatment, cells were fixed with 3% ice-cold paraformaldehyde for 10 min at room temperature (RT, 22–24 °C), washed with PBS and blocked/permeabilised with PBS containing 5% bovine serum albumin (BSA) and 0.2% Triton X-100 for 1 h at RT. Cells were subsequently incubated for 14–16 h at 4 °C with the following primary antibodies: mouse anti PIP_2_ (2C11, dil 1:200, Santa Cruz, Dallas, USA) and a loading control rabbit anti-cytochrome c oxidase subunit IV (COX-IV, dil 1:1000, Abcam, Cambridge, UK). After three washes with PBS (10 min each), cells were incubated with anti-mouse and anti-rabbit IgG conjugated to IRDye® 680RD and IRDye® 800CW, respectively (dil 1:1000, Li-Cor, Cambridge, UK), for 1 h at RT. All antibodies were diluted in PBS containing 1% BSA and 0.04% Triton X-100. Cells were then washed three times with PBS and imaged on the Odyssey Infrared Imaging System (Li-Cor, Cambridge, UK) and analysed with supplier’s software (Version 3.1).

### Statistical analysis

All data are mean ± s.e.m. of *n* cells. One-way ANOVA test followed by a Dunnett’s or Tukey’s multiple comparisons test or Student’s *t* test were used to determine statistical significance between groups, where * = *P* < 0.05, ** = *P* < 0.01, *** = *P* < 0.001 and **** = *P* < 0.0001.

### Reagents

Many different pharmacological tools were used to either alter PIP_2_ levels or impair βγ G protein interactions. These are listed below with the supplier and mechanism of action.

### PIP_2_ depletion

Wortmannin (Sigma Aldrich, Poole, UK), at 20 μM, is an inhibitor of myosin light chain kinase, phosphatidylinositol 3-kinase and phosphatidylinositol 4-kinase. It depletes PIP_2_ levels by inhibiting synthesis from phosphatidyl inositol via the phosphatidylinositol 4-kinase [1, 21, 28]. PIP_2_ depletion was augmented by brief application of 1 μM trypsin that activates protease-activated receptors endogenous to the HEK cell [30]. In addition, the phospholipid acceptor α-Cyclodextrin and LY-294,002 hydrochloride, another inhibitor of phosphatidylinositol kinase (both from Sigma), were used for in-cell western blot studies.

### Prevention of βγ G protein interaction

We used a range of structurally different compounds that prevent βγ G proteins interacting with effector proteins through different mechanisms. Gβγ-target recognition is defined by a single “hotspot,” which has distinct sub-surfaces for individual G protein subunit interactions [3]. Gallein (Tocris, Avonmouth, UK), M201 and M119K (provided by National Cancer Institute Drug Development Programme, 3), all bind to the hot spot at concentrations less than 1 μM but differentially modulate Gβγ interactions with effectors [3]. Grk2i (Tocris, Avonmouth, UK, 10 μM) is a peptide analogue of the G-protein receptor kinase c-terminus [16] which competes with effector proteins for βγ G protein binding. All reagents were applied to the bathing solution at concentrations derived from previous publications.

### Additional materials

G-protein βγ subunits from bovine brain were purchased from Merck. PIP_2_ (d-*myo*-phosphatidylinositol 4,5-bisphosphate) was purchased from Echelon. HEK293 cells stably expressing Kv7.4 were a gift from the University of Copenhagen [2, 24]. Retigabine, S-1, and NS15370 were synthesized by NeuroSearch A/S, (Ballerup, Denmark). The pan-Kv7 blocker linopirdine was purchased from Tocris (Avonmouth, UK).

## Results

### PIP_2_ depletion abolishes Kv7.4 currents and prevents their activation by Gβγ

**Fig. 1:**
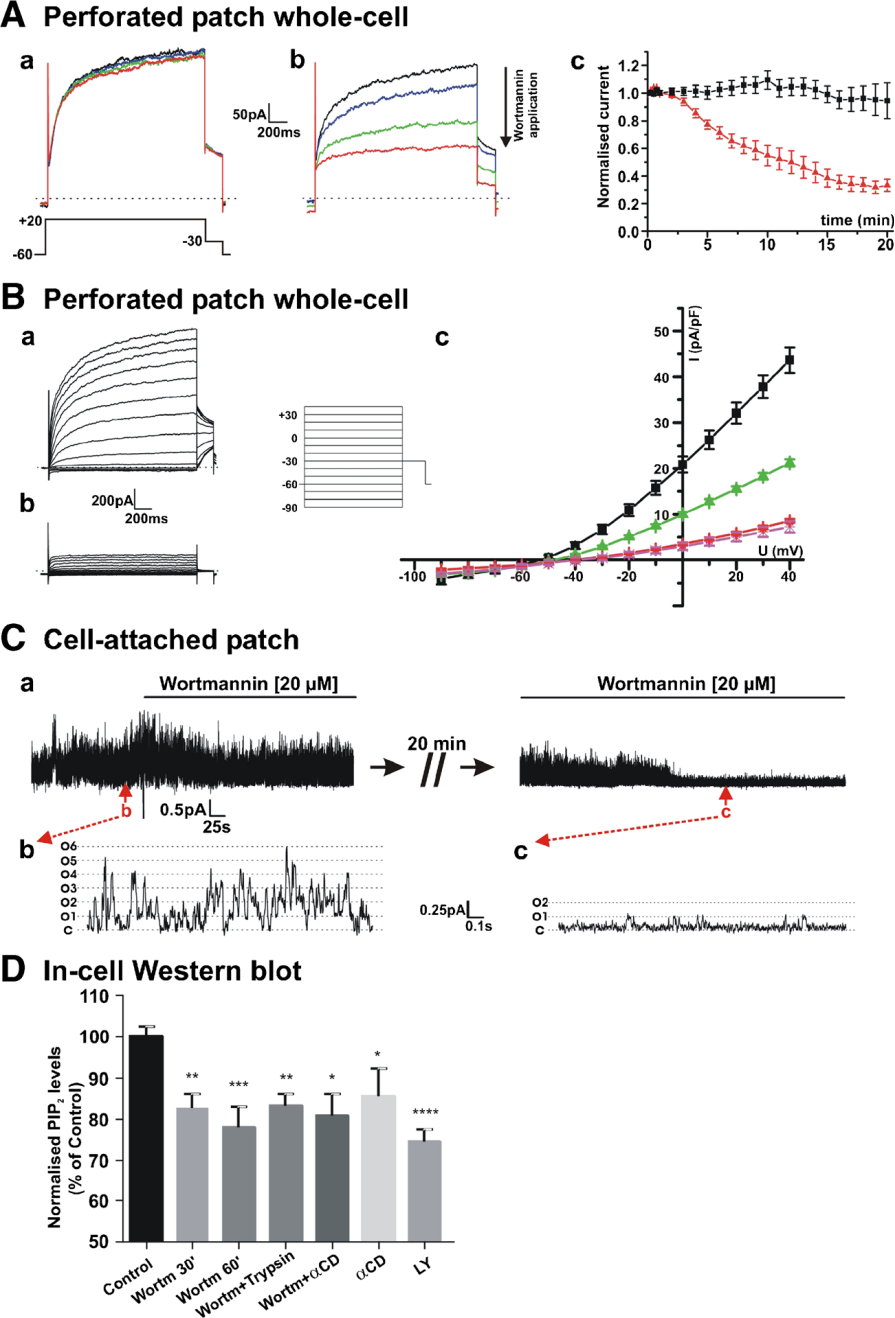
PIP_2_ depletion reduces Kv7.4 currents. **A** Examples of whole cell K^+^ currents from HEK293 Kv7.4 cells evoked by step depolarisation from −60 mV to +20 mV in the absence (*a*) and presence of 20 μM wortmannin (*b*). Currents were recorded every 15 s and wortmannin applied after 60 s. Initial current trace is shown in *black* in both subpanels. Subsequent traces after 5-, 10- and 20-min intervals are shown in *blue*, *green* and *red*, correspondingly. Subpanel *c* shows the mean amplitude of K^+^ current at +20 mV in the absence (*black*) and presence of wortmannin (*red*). Each *point* is the mean ± s.e.m. of four cells. **B** Representative traces of Kv7.4 currents evoked by steps from −60 mV to a range of potential (−90 to +40 mV) in control (*a*) and after depletion of PIP_2_ by the cells preincubation with wortmannin + short (≤30 s) application of trypsin (*b*). The mean data are shown in subpanel *c* with control (*black*, *n* = 22), wortmannin alone (*green*, *n* = 34), wortmannin plus trypsin (*red*, *n* = 36) and linopirdine (*purple*, *n* = 14). **C** Example of cell-attached patch recording from HEK293 Kv7.4 cell showing the effect of 20 μM wortmannin. Long-term trace is shown in subpanel *a*. Representative expanded 1.75-s segments of channel openings taken from subpanel *a* highlighting channel activity in the absence, and the presence of wortmannin are shown in subpanels *b* and *c*. Closed state and multiple open states are denoted by *C* and *O1–O6*. **D** In-cell Western analysis showing that wortmannin, and other known PIP_2_ inhibitors, decrease global PIP_2_ level in HEK293 Kv7.4 cells (*n* = 12–23)

**Fig. 2:**
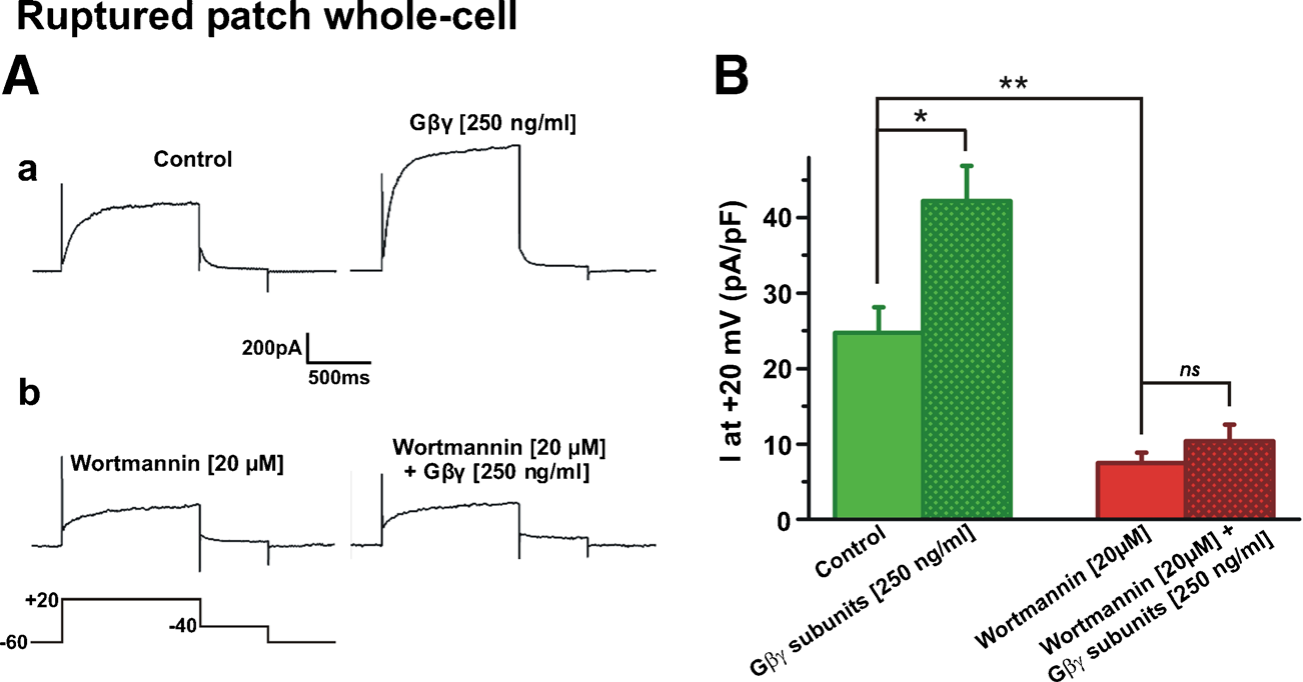
PIP_2_ depletion prevents activation of whole-cell Kv7.4 currents by Gβγ. **A** Representative whole-cell currents evoked by depolarisation from −60 to +20 mV in Kv7.4 HEK293 cells under control conditions (*a*) and after incubation in wortmannin (20 μM, *b*). *Right-hand panels* show cells perfused internally with Gβγ. **B** Mean data for the effect of intracellular perfused active Gβγ on whole-cell currents recorded at +20 mV in the absence and presence of 20 μM wortmannin (*n* = 5)

**Fig. 3:**
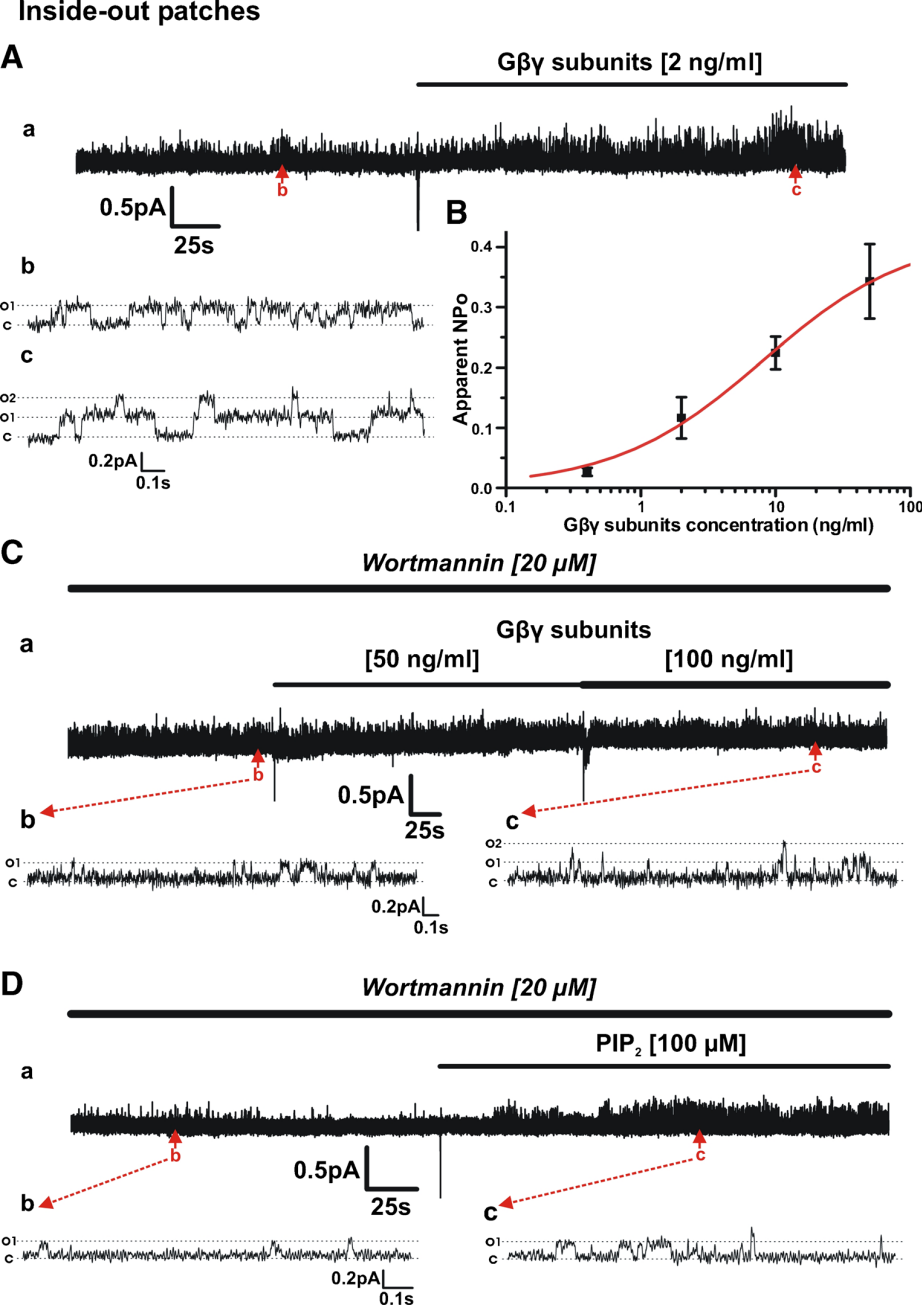
Gβγ enhancement of Kv7.4 channels in excised patches is prevented by PIP_2_ depletion. **A** Representative inside-out patch recording showing stimulatory action of Gβγ. Expanded 1.75 s segments of channel openings in the absence (*b*) and presence of 2 ng/ml Gβγ (*c*) are taken from long-term recording (*a*). Closed state and multiple open states are denoted by *C* and *O1–O2*. **B** Mean concentration effect for Gβγ subunits (*n* = 4–7) fitted with a best-fit sigmoidal to the available data. **C** Representative inside-out patch recording from cell preincubated with wortmannin before (*b*) and after (*c*) Gβγ application. Subpanels *b* and *c* are expanded 2.5-s segments of channel openings taken from long-term recording (*a*). Closed state and multiple open states are denoted by *C* and *O1–O2*. **D**, Representative inside-out patch recording from cell preincubated with wortmannin before (*b*) and after (*c*) PIP_2_ application. Subpanels *b* and *c* are expanded 1.35-s segments of channel openings taken from long-term recording (*a*). Closed state and open states are denoted by *C* and *O1*

In HEK cells stably expressing Kv7.4, depolarisation evoked characteristic time-dependent currents (Fig. 1) that were abolished by treatment with the Kv7 blocker linopirdine (10 μM) and were not apparent in untransfected HEK cells [27]. Application of 20 μM wortmannin, to reduce PIP_2_ levels, gradually decreased Kv7.4 currents recorded in perforated-patch whole-cell configuration (Fig. 1A). Further inhibition of currents to a level identical to that recorded after application of the Kv7 channel blocker linopirdine (10 μM) was achieved by brief (≤30 s) application of 1 μM trypsin to stimulate endogenous G-protein-coupled proteinase-activated receptors (Fig. 1B). Wortmannin also reduced Kv7.4 channel activity in cell-attached experiments (Fig. 1C). In-cell western analysis showed that wortmannin alone and in the combination with trypsin application significantly decreased levels of PIP_2_ in Kv7.4-transfected HEK cells (Fig. 1D). We then investigated whether PIP_2_ depletion modified the stimulatory response to enrichment of internal solutions with Gβγ. Like our previous study [27], intracellular perfusion of active Gβγ (250 ng/ml) increased current amplitude by ~70% within 5 min of rupture in control cells (Fig. 2A (a), B) but had considerably less effect (~38% increase) in cells incubated with 20 μM wortmannin (Fig. 2A (b), B). We then undertook inside-out excised patch recording to investigate this effect further. In HEK cells stably expressing Kv7.4 but not untransfected cells, robust K^+^ channel activity was recorded immediately upon patch excision that usually decayed within 1–2 min to a steady, lower level of activity. This is considered to be due to the wash out of key intracellular mediators from the membrane patch [4, 12, 18]. Bath application of Gβγ (0.4–50 ng/ml) increased channel activity in a concentration-dependent manner similar to previous work [27] with an estimated value for half maximal stimulation of 8.1 ng/ml (*n* = 4–7, Fig. 3A, B). However, bath application of Gβγ had no effect (2.51 ± 0.05% increase only, *n* = 4) on the negligible channel activity recorded in the continued presence of wortmannin (Fig. 3C) whereas application of exogenous PIP_2_ in the continued presence of wortmannin re-established channel activity (Fig. 3D,*n* = 5). These data show that inhibition of PIP_2_ re-synthesis by wortmannin decreased PIP_2_, reduced channel activity and prevented Gβγ-mediated channel stimulation suggesting that Gβγ may act upstream of PIP_2_.

### Gβγ inhibition abolishes Kv7.4 currents and prevents their activation by PIP_2_

**Fig. 4:**
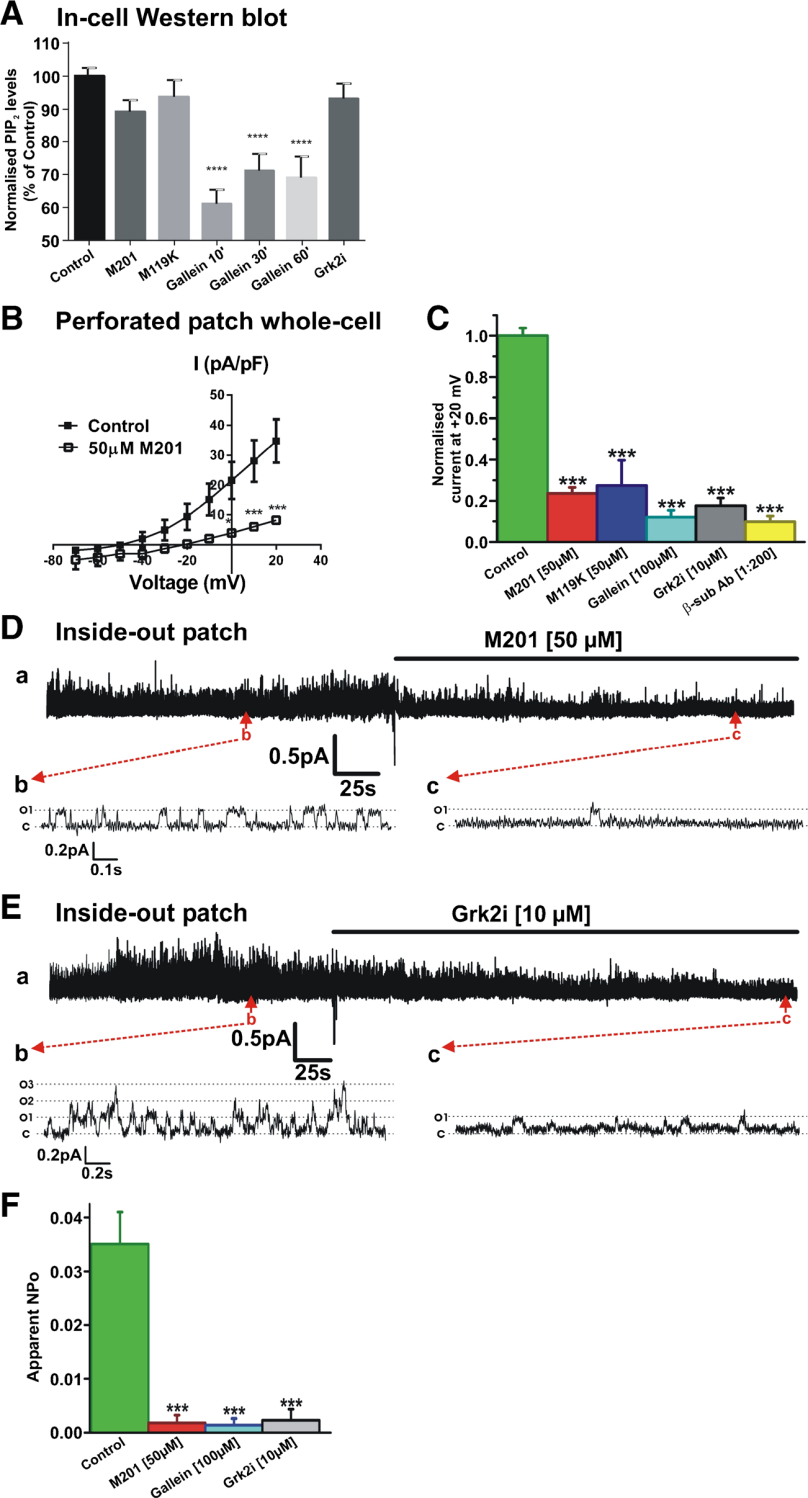
Gβγ inhibition abolishes Kv7.4 currents without change in PIP_2_ levels**. A** In-cell Western analysis showing influence of Gβγ inhibitors on PIP_2_ level in HEK293 Kv7.4 cells (*n* = 11–22). **B** Mean I-V relationships of HEK293 Kv7.4 currents evoked from holding potential −60 mV before and after application of M201 (50 μM). **C** Mean data for the effect for mechanistically different inhibitors of Gβγ on whole-cell K^+^ currents recorded at +20 mV (*n* = 5–7). **D** Representative inside-out patch recording showing inhibitory action of 50 μM M201 on single channel activity. Subpanels *b* and *c* are representative 1.5-s segments of channel openings before and after drug application taken from long-term recording (*a*). Closed and open states are denoted by *C* and *O1*. **E** Representative inside-out patch recording showing inhibitory action of 10 μM Grk2i on single channel activity. Subpanels *b* and *c* are expanded 2.8-s segments of channel openings before and after drug application taken from long-term recording (*a*). Closed state and multiple open states are denoted by *C* and *O1*–*O3*. **F** Mean apparent open probability for Kv7.4 channels in control conditions (green column, *n* = 14) and after application of three different Gβγ inhibitors, M201, gallein and Grk2i (*n* = 4–6)

Having established that PIP_2_ depletion impaired Gβγ-mediated enhancement of Kv7.4 currents, we addressed whether reduction of Gβγ activity limited the well-established PIP_2_ enhancement of channels. Initially, we used the in-cell western blot technique to ascertain whether Gβγ inhibitors altered PIP_2_ levels. Figure 4A shows that neither Grk2i, M201 nor M199K altered PIP_2_ levels whilst the well-used small molecule Gβγ inhibitor gallein reduced PIP_2_ to some extent. In a previous study, we showed that gallein, Grk2i and an antibody raised against Gβ inhibited whole-cell Kv7.4 currents markedly [27]. Application of two novel and potent Gβγ inhibitors, M201 and M119K [3], also inhibited whole-cell Kv7.4 currents significantly (Fig. 4B, C). Figure 4C shows the accumulated data for all Gβγ inhibitors on whole-cell Kv7.4 currents highlighting the suppressive effect irrespective of mechanistic action. Having established the effect of Gβγ inhibitors on whole-cell currents, we then performed inside-out recordings to determine how single-channel activity was affected. Figure 4D, and E shows that M201 and Grk2i reduced the open probability of Kv7.4 channels in excised patches to negligible levels similarly to the action of gallein (Fig. 4F) [27].

**Fig. 5:**
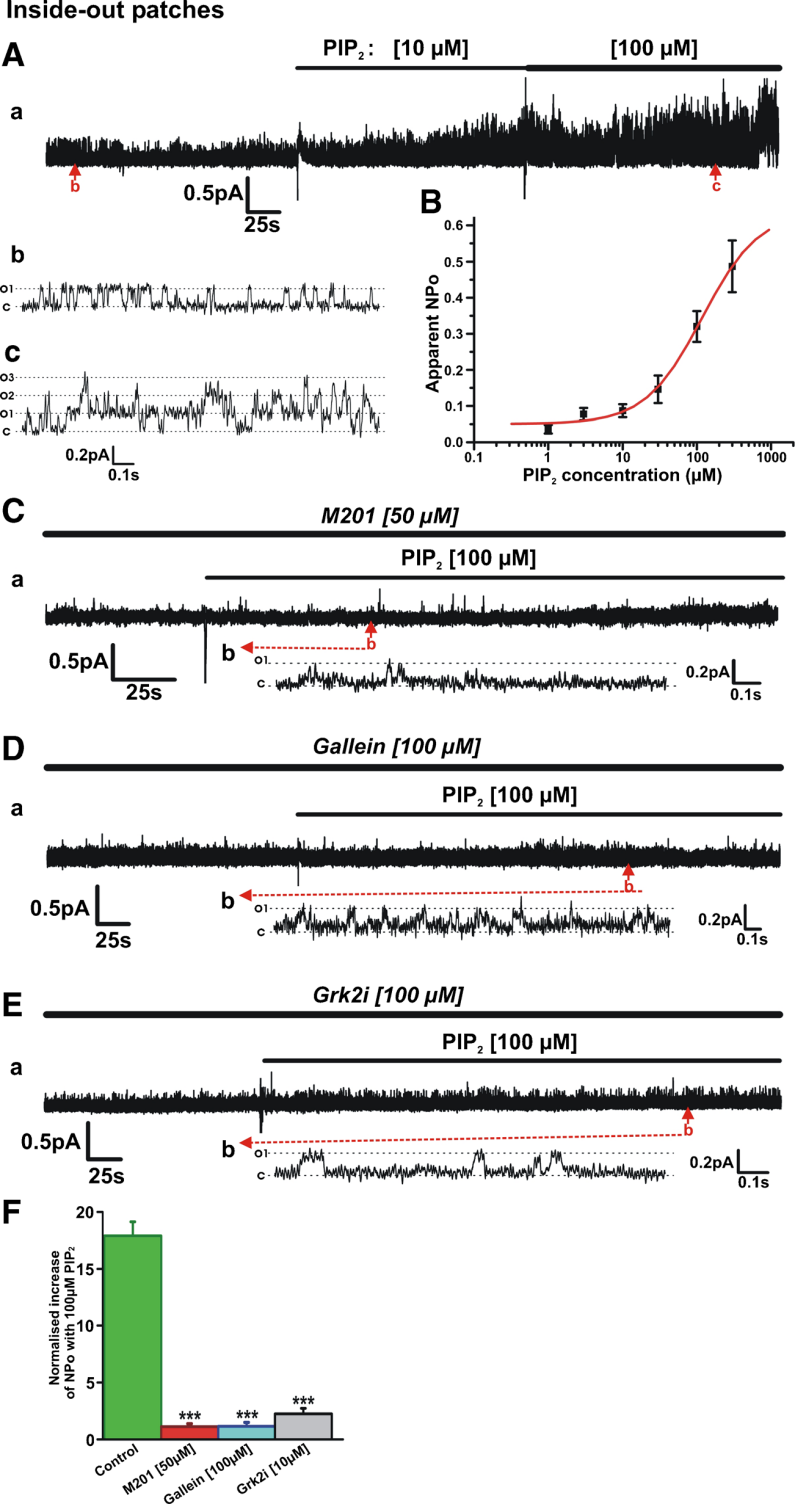
Gβγ inhibition prevents activation of Kv7.4 channels by exogenous PIP_2_. **A** Representative inside-out patch recording showing stimulatory action of PIP_2_. Subpanels *b* and *c* are 1.75-s segments of channel openings in the absence (*b*) and presence of PIP_2_ (100 μM, *c*) taken from long-term recording (*a*). Closed state and multiple open states are denoted by *C* and *O1*–*O3*. **B** Mean concentration-effect for PIP_2_ (*n* = 4–11). **C** Representative inside-out patch recording showing that in the continued presence of 50 μM M201 (continuation of recording from patch shown in Fig. 4D), 100 μM PIP_2_ applied to the patch failed to activate the channels. Subpanel *b* is a 1.5-s segment of channel openings taken from long-term recording (*a*). Closed state and multiple open states are denoted by *C* and *O1*. **D** Representative inside-out patch recording showing that in the continued presence of 100 μM gallein, 100 μM PIP_2_ applied to the patch failed to activate the channels. Subpanel *b* is a 2.5-s segment of channel openings taken from long-term recording (*a*). **E** Representative inside-out patch recording showing that in the continued presence of 100 μM Grk2i (continuation of recording from patch shown in Fig. 4E), 100 μM PIP_2_ applied to the patch produced only negligible activation of the channels. Subpanel *b* is a 2.8-s segment of channel openings taken from long-term recording (*a*). **F** Normalized increase in NPo produced by 100 μM PIP_2_ applied to inside-out patches in the absence and presence of different Gβγ inhibitors (*n* = 4–6)

Having confirmed that inhibition of Gβγ inhibited Kv7.4 currents recorded at the whole-cell and single-channel level, we used inside-out excised patches to ascertain if structurally different Gβγ inhibitors altered PIP_2_-induced enhancement of Kv7.4 activity. Under control conditions, application of PIP_2_ increased the apparent channel open probability (NPo) of the Kv7.4 channel activity in a concentration-dependent manner (Fig. 5A, B). This resulted in an estimated value for half-maximal stimulation of 117 μM (*n* = 4–11) similar to previous papers [4, 12, 18]. By comparison, no significant increase in Kv7.4 channel activity was observed upon application of PIP_2_ (10–100 μM) in patches incubated in M201, gallein or Grk2i (Fig. 5C–F). These data reveal that inhibition of Gβγ interaction by structurally and mechanistically different agents not only inhibits Kv7.4 basal activity but also prevented PIP_2_ stimulation of Kv7.4 (Fig. 5F).

### PIP_2_ and Gβγ are synergistic regulators of Kv7.4 channels

**Fig. 6:**
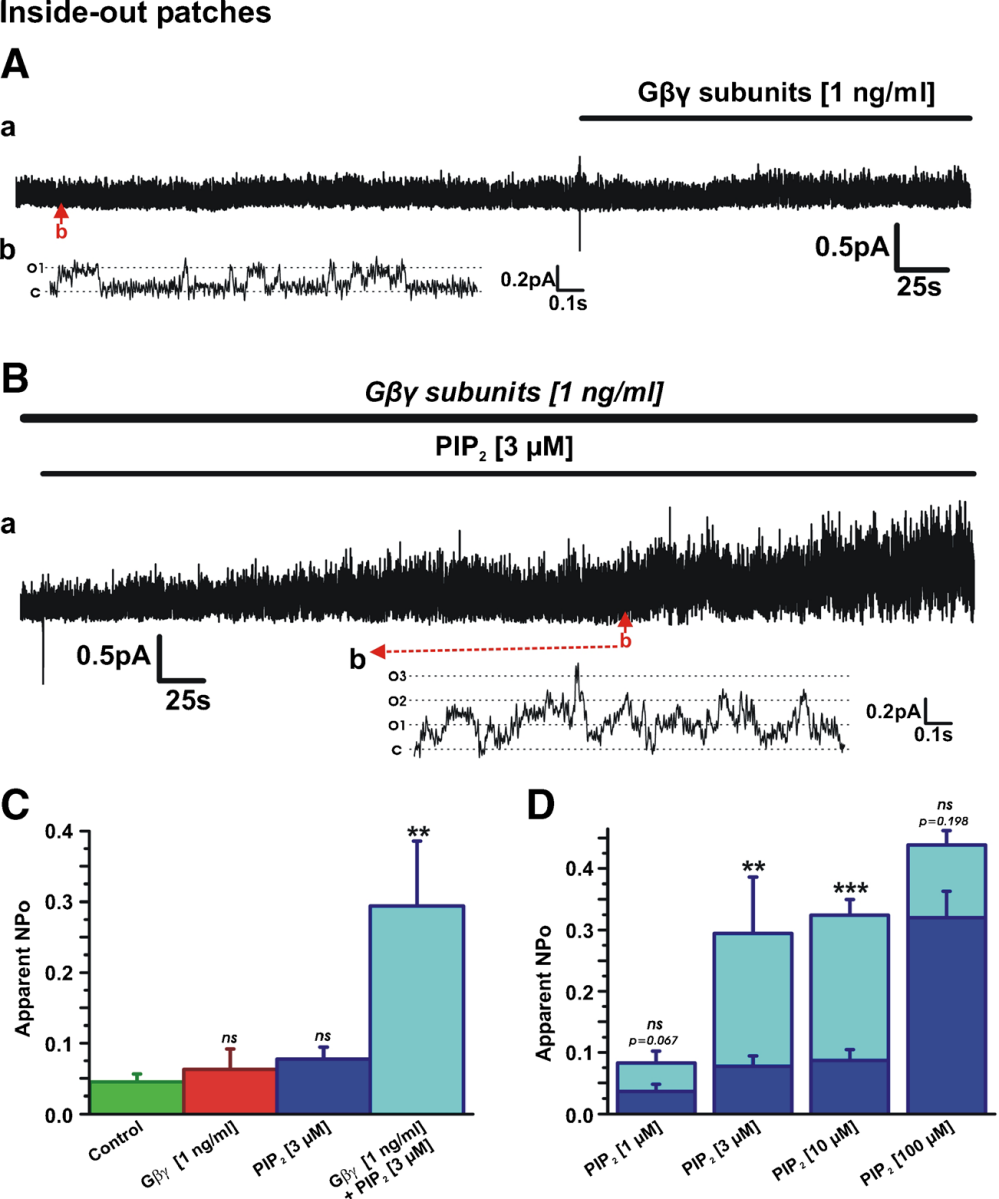
PIP_2_ and Gβγ are synergistic regulators of Kv7.4 channels**. A** Representative inside-out patch recording showing lack of effect of 1 ng/ml Gβγ on channel activity. Subpanel *b* is an expanded 1.65-s segment of channel openings taken from long-term recording (*a*). Closed and open states are denoted by *C* and *O1*. **B** Application of 3 μM PIP_2_ in the presence of 1 ng/ml Gβγ (patch from (**A**)) significantly increased channel activity. Subpanel *b* is an expanded 1.65-s segment of channel openings taken from long-term recording (*a*). Closed state and multiple open states are denoted by C and *O1–O3*. **C** Mean apparent open probability for Kv7.4 in control conditions (*n* = 16), after application of low concentrations of Gβγ (*n* = 8) and PIP_2_ alone (*n* = 8) and in combination (*n* = 6). **D** Mean apparent open probability for Kv7.4 activated by PIP_2_ alone (*n* = 4–11, *dark blue columns*) and in the presence of 1 ng/ml Gβγ (*cyan columns*, *n* = 4–10)

Our data thus far suggest that rather than regulation of Kv7.4 by PIP_2_ and Gβγ being a linear relationship, the signal molecules combine to dictate channel function. The next series of inside-out experiments aimed to determine if a low sub-effective concentration of Gβγ could enhance the sensitivity of the channel to exogenous PIP_2_. Application of low concentrations of either Gβγ (1 ng/ml) or PIP_2_ (1–3 μM) to inside-out patches had a negligible effect on Kv7.4 channel activity (Fig. 6A, C ). However, Fig. 6B–D shows that in combination, a marked increase in channel activity was observed and the presence of 1 ng/ml Gβγ increased the sensitivity of the channel to PIP_2_. For instance, NPo increased to 0.29 ± 0.07 (*n* = 6) when 3 μM PIP_2_ was applied in the presence of 1 ng/ml Gβγ, which was significantly greater than NPo when 3 μM of PIP_2_ alone was applied (0.077 ± 0.02, *n* = 8; Fig. 6C). The stimulatory effect of Gβγ was not apparent when 100 μM PIP_2_ was applied, suggesting that the combinational effect had a functional ceiling (Fig. 6D). Consequently, low concentrations of Gβγ produced a leftward shift in the sensitivity of the Kv7.4 channel to PIP_2_ suggesting the two molecules acted synergistically.

### PIP_2_ depletion and reduced Gβγ activity affects pharmacological modulation of Kv7.4

**Fig. 7:**
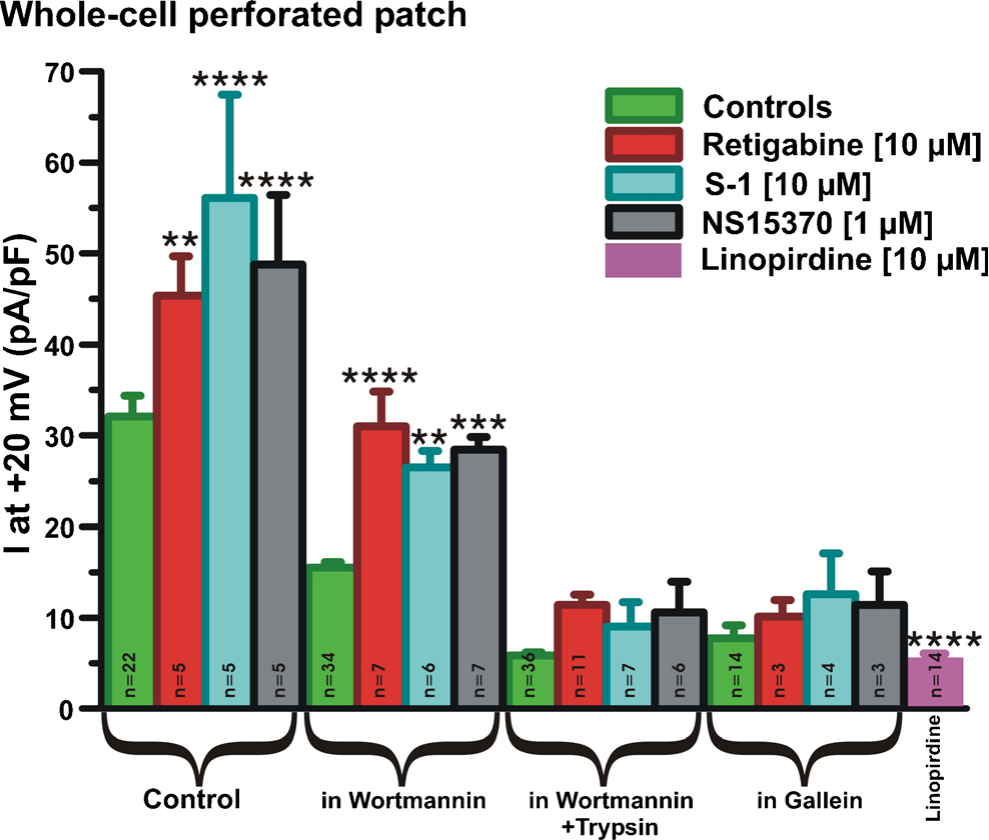
Effect of Kv7 channel openers in PIP_2_- and Gβγ-depleted cells. Mean data showing action of Retigabine, S-1 and NS15370 in HEK293 Kv7.4 cells under various conditions (control, preincubated with wortmannin alone, preincubated with wortmannin + short (≤30 s) application of trypsin, preincubated with gallein). Currents after treatment with pan-Kv7 channel blocker linopirdine are shown for reference. *, **, *** and **** denote *p* < 0.05 – *p* < 0.0001, respectively, compared to controls

Application of three structurally different enhancers of Kv7.2–7.5, S-1, retigabine and NS15370, produced a marked increase in whole-cell currents in HEK cells stably expressing Kv7.4 with currents at +20 mV increasing by approximately 40% (Fig. 7). In cells treated with wortmannin alone, some enhancement with each activator was observed. However, in cells bathed in either wortmannin plus trypsin or gallein alone, the stimulatory effect of all three agents was abrogated. Consequently, the ability of pharmacological agents to augment Kv7.4 was compromised by PIP_2_ depletion or reduced βγ subunit activity.

## Discussion

It is accepted dogma that Kv7 channels have an obligatory reliance on PIP_2_ for effective function. We now reveal that this positive regulation occurs synergistically with Gβγ, and there is an obligatory reliance on both mediators to be present for effective channel function. These findings change radically our understanding on how Kv7.4 channels are regulated. If other Kv7 channels are also affected by Gβγ in a similar manner, the findings of the present study will have wide-ranging implications as Kv7 channels regulate cellular physiology in many cell types. We show by in-cell western blot that treatment with the phosphatidylinositol-4-kinase inhibitor wortmannin and stimulation of G-protein coupled receptors with trypsin reduced global PIP_2_ and reduced Kv7.4 currents to negligible levels (Fig. 1). More importantly, this treatment abrogated the stimulatory effect of Gβγ on Kv7.4 channels but did not impair the ability of exogenous 100 μM PIP_2_ to enhance Kv7.4 channel activity in excised patches (Fig. 3). This implied PIP_2_ sensitization underlies the positive effect of Gβγ. However, treatment with structurally and mechanistically different inhibitors of Gβγ interactions (gallein, M201, M199K and Grk2i) also decreased Kv7.4 channel activity (Figs. 3 and 4) to the same levels as treatment with wortmannin and trypsin, which mirrored the inhibition produced by the Kv7 channel blocker linopirdine. Moreover, all Gβγ inhibitors prevented any increase in channel activity by exogenous PIP_2_ (100 μM) in excised patches (Fig. 5). It is worth stressing that these inhibitors disable Gβγ interactions with target proteins through different binding domains and mechanisms [3], consistent with Gβγ having differential protein effector sites but all suppressed PIP_2_-induced enhancement of Kv7.4 open probability. These findings suggested that PIP_2_ was not simply an upstream channel regulator. In fact, these findings reveal that inherent Kv7.4 channel activity is dictated by a coordinated interaction with PIP_2_ and Gβγ. Removal of either regulatory arm leads to progressive run down of channel activity and refractoriness to stimulation by the opposing molecule. The synergistic effect of PIP_2_ and Gβγ was substantiated by the observation that a sub-efficacious dose of Gβγ (1 ng/ml) potentiated the response to a low concentration of PIP_2_ (1–10 μM), but not a saturating dose of PIP_2_ (100 μM), resulting in a pronounced leftward shirt in the response to exogenous PIP_2_ (Fig. 6).

In addition to the control of Kv7.4 activity at rest, we also show that compounds that activate Kv7.2–7.5 through a common molecular mechanism [14, 17, 22, 33] also required PIP_2_ and Gβγ binding to be effective. These findings corroborate previous work that showed retigabine-induced stimulation of Kv7.2/7.3, which comprise the neuronal M-channel, was negligible after PIP_2_ depletion [35]. Interestingly, the pharmacological dependence on PIP_2_ was localized to an interaction site more proximal than the well-established C-terminus site necessary for channel activity [35]. It is possible that the synergistic effect of Gβγ is due to alterations of endogenous levels of PIP_2_ as Gβγ do activate phospholipase Cβ and phosphoinositide-3-kinase [23]. However, these enzymes would reduce rather than enhance PIP_2_ levels. Moreover, the Gβγ inhibitors M201, M199K and Grk2i had no discernible effect on PIP_2_ levels whereas gallein decreased global PIP_2._ This effect of gallein has not been reported previously and there is no reason for the effect. The fact that other Gβγ inhibitors did not alter global PIP_2_ levels suggests it is a quirk of the molecule rather than of Gβγ inhibition.

The dual regulation of Kv7.4 by PIP_2_ and Gβγ identified in the present study bears a considerable similarity to GIRK channels, which have a co-dependence on Gβγ and PIP_2_ [10, 20]. Early studies proposed that Gβγ stabilized the PIP_2_ interaction with Kir3.1/Kir3.4 [13, 29]. Crystallographic studies revealed that full GIRK activation was reliant upon PIP_2_ interacting with an internal gate independent of the G-gate where Gβγ binds, and no channel openings occurred if either gate was unoccupied [31, 32]. Currently, the same level of molecular insight does not exist for Kv7.4. PIP_2_-binding domains have been identified in other Kv7 family members, but this information is lacking for Kv7.4. In addition, there is no information on binding sites for Gβγ. In GIRK channels, approximately 12 sites across the protein have been identified as important for Gβγ binding that combine to accommodate 4 Gβγ molecules in the functional tetramer [9, 11, 34]. Moreover, distinct high- and low-affinity sites exist in GIRKs that determine basal activation and receptor-mediated activation, respectively [11]. These aspects of molecular recognition need to be determined for Kv7.4.

A criticism that could be levelled at this study is it relies solely on pharmacological agents for the conclusion. However, we use blockers that are not only structurally different but which work through varied mechanisms. As wortmannin and various inhibitors of Gβγ reduced Kv7.4 channel activity to negligible levels, there must be sufficient PIP_2_ and Gβγ in the channel locality to sustain channel activity under excised patch conditions. It is generally assumed that PIP_2_ levels remain consistent in a normal healthy cell and are replenished rapidly upon hydrolysis. Our data show that Kv7.4 activity is attenuated by Gβγ inhibitors, implying that there is a persistent influence of Gβγ maintaining channel activity. Again, there is a parallel with GIRK channels that have a basal level of activity due to constitutive binding of Gβγ [11]. The free Gβγ maintaining Kv7.4 activity may be the product of binding of Gα to the channel protein and the associated tethering of Gβγ as shown for Kir3.1 [11], localized G-protein coupled receptor activity or a labile free pool of Gβγ. Irrespective of these unknowns, we have identified that Kv7.4 activity is crucially dependent on a synergistic interplay between PIP_2_ and Gβγ. Kv7 channels are well known to have an obligatory requirement for PIP_2_ but this study reveals a more complex and subtle paradigm where the reliance on local phosphoinositide is dictated by an involvement of Gβγ. Whilst the present study focuses on heterologous expression, the findings have physiological implications because Kv7 channel isoforms have a key role in many cell types. Kv7.1 comprises the late repolarising component of the cardiac action potential, Kv7.2/7.3 and Kv7.5/7.3 heteromers constitute the M-channel in neurones and Kv7.4 has a role in cochlear as well as arterial reactivity. Dysregulation of these channels impacts considerably on cellular activity in each cell type under consideration. Defining the mechanisms that regulate Kv7 channels is therefore paramount for understanding physiological and pathophysiological processes. We have already established that βγ G proteins modulate endogenous Kv7 channels in arterial smooth muscle cells consistent with their effects on heterologously expressed Kv7.4 [24], and it is likely that similar effects occur on native Kv7 channels in other systems. If this is the case, then revelation of the present study will have profound resonance for cellular regulation.
